# Apomorphine

**Published:** 1888-02

**Authors:** 


Apomorphine.—The value of this agent, as a prompt and certain emetic, has been generally recognized. A serious objection to its use is the rapidity with which its aqueous solution changes color, and the authorities generally have declared that in this condition it loses its medicinal powers, and should not be used. Dr. Murrell, of London, however, affirms that this is an error; that the development of color does not injure it in any way, and that the solution may be kept for years, and is safe to use hypodermically.

---

THE annual meeting of the Erie County Society took place on the 10th ult. Dr. John D. Hill was elected president for the ensuing year, and Dr. R. L. Banta vice-president. The official report of the proceedings was not received in time for publication in this number.

---

DR. F. S. CREGO, Professor of Nervous and Mental Diseases, in Niagara University, Medical Department, has removed to his new house, 280 Franklin street, which he built last summer, and which is a model, and well adapted to his business. We wish our friend continued prosperity in new surroundings.

